# A Histone Deacetylase Inhibitor Induces Acetyl-CoA Depletion Leading to Lethal Metabolic Stress in RAS-Pathway Activated Cells

**DOI:** 10.3390/cancers14112643

**Published:** 2022-05-26

**Authors:** Agnes Basseville, Pierre-Christian Violet, Maryam Safari, Carole Sourbier, W. Marston Linehan, Robert W. Robey, Mark Levine, Dan L. Sackett, Susan E. Bates

**Affiliations:** 1 Developmental Therapeutics Branch, National Cancer Institute, National Institutes of Health, Bethesda, MD 20892, USA; robeyr@mail.nih.gov; 2 Omics Data Science Unit, Institut de Cancérologie de l’Ouest, 49055 Angers, France; 3 Molecular and Clinical Nutrition Section, National Institute of Diabetes and Digestive and Kidney Diseases, National Institutes of Health, Bethesda, MD 20892, USA; pierre-christian.violet@nih.gov (P.-C.V.); mark.levine@nih.gov (M.L.); 4 Division of Hematology/Oncology, Department of Medicine, Columbia University Medical Center, New York, NY 10032, USA; ms5244@cumc.columbia.edu; 5 Urology Oncology Branch, National Cancer Institute, National Institutes of Health, Bethesda, MD 20892, USA; carole.sourbier@fda.hhs.gov (C.S.); linehanm@mail.nih.gov (W.M.L.); 6 Office of Biotechnology Products, Office of Pharmaceutical Quality, Center for Drug Evaluation and Research, U.S. Food and Drug Administration, Silver Spring, MD 20903, USA; 7 Laboratory of Cell Biology, National Cancer Institute, National Institutes of Health, Bethesda, MD 20892, USA; 8 Division of Basic and Translational Biophysics, Eunice Kennedy Shriver National Institute of Child Health and Human Development, National Institutes of Health, Bethesda, MD 20892, USA; sackettd@mail.nih.gov; 9 Hematology/Oncology Research Department, James J. Peters Department of Veterans Affairs Medical Center, New York, NY 10468, USA

**Keywords:** HDAC inhibitors, acetyl-CoA metabolism, RAS

## Abstract

**Simple Summary:**

Epigenetic therapies have been difficult to translate to solid tumors, in part because a lack of a full mechanistic understanding has not allowed the selection of tumors most likely to benefit from the therapies. Here we identified a RAS-phenotype that can be targeted by the histone deacetylase inhibitor (HDACi) romidepsin. We showed that the hyperacetylation induced by romidepsin depletes acetyl-CoA, the cell donor substrate for acetylation, and it leads to metabolic stress and death in KRAS-activated cells. Transcriptomic analysis confirmed that perturbation of two acetyl-CoA generation pathways (fatty acid metabolism and branched-chain amino acid (BCAA) metabolism) were correlated with HDACi sensitivity in a 608-cell line panel and in patients treated with belinostat. Our analysis highlights the potential utility of an acetyl-CoA phenotype to sharpen treatment choices for RAS-activated tumors, and it suggests that acetyl-CoA depletion could be a key effect underlying the myriad cellular responses that follow HDAC inhibition.

**Abstract:**

Background: The mechanism of action of romidepsin and other histone deacetylase inhibitors is still not fully explained. Our goal was to gain a mechanistic understanding of the RAS-linked phenotype associated with romidepsin sensitivity. Methods: The NCI60 dataset was screened for molecular clues to romidepsin sensitivity. Histone acetylation, DNA damage, ROS production, metabolic state (real-time measurement and metabolomics), and gene expression alterations (transcriptomics) were determined in KRAS-WT versus KRAS-mutant cell groups. The search for biomarkers in response to HDACi was implemented by supervised machine learning analysis on a 608-cell transcriptomic dataset and validated in a clinical dataset. Results: Romidepsin treatment induced depletion in acetyl-CoA in all tested cell lines, which led to oxidative stress, metabolic stress, and increased death—particularly in KRAS-mutant cell lines. Romidepsin-induced stresses and death were rescued by acetyl-CoA replenishment. Two acetyl-CoA gene expression signatures associated with HDACi sensitivity were derived from machine learning analysis in the CCLE (Cancer Cell Line Encyclopedia) cell panel. Signatures were then validated in the training cohort for seven HDACi, and in an independent 13-patient cohort treated with belinostat. Conclusions: Our study reveals the importance of acetyl-CoA metabolism in HDAC sensitivity, and it highlights acetyl-CoA generation pathways as potential targets to combine with HDACi.

## 1. Introduction

RAS-mutated tumors are the most common and the deadliest of all human cancers, and they remain among the most refractory to available treatments [1,2]. In the wild-type (WT) state, RAS protein activation/deactivation controls central cellular processes such as growth, migration, or survival, as well as interactions with cells from the extracellular environment. When mutated, RAS remains in its active state, and it continuously activates its downstream effectors (RAF, PI3K, RALGDS, and TIAM1) leading to aberrant signaling in these pathways [1]. 

Interestingly, our group and others [3,4,5] have shown that RAS-transformed cells display enhanced sensitivity to the histone deacetylase (HDAC) class I inhibitor romidepsin, although the mechanism of this sensitivity has not yet been elucidated. HDAC inhibitors (HDACis) are epigenetic modulators initially known to induce hyperacetylation of histone proteins leading to transcriptional deregulation. Other biological consequences of HDAC inhibitors have also been demonstrated, including accumulation of RNA-DNA hybrids known as R-loops, DNA damage, oxidative stress, metabolic perturbations, cell cycle arrest, and apoptosis [6,7,8]. It was approved by the U.S. Food and Drug Administration in 2009 to treat T-cell lymphoma, but it has limited effect in monotherapy for solid tumors. Its pleiotropic mechanism of action remains of high interest in the scientific community in finding appropriate drug combinations for solid tumors in the clinic [6]. 

Apart from new G12C genotype targeted therapies, strategies to kill RAS-mutant cells by directly targeting RAS or its downstream effectors have been mostly unsuccessful, mainly due to pathway redundancy and heterogeneities in RAS-induced phenotypes. One therapeutic strategy that has emerged involves the exploitation of tumor metabolic vulnerabilities induced by RAS activation [9]. In fact, cancer cells rewire their metabolic pathways to meet their needs in energy and biosynthetic precursors, both essential for unrestrained proliferation. For example, in pancreatic cancer, KRAS promotes a shift in the glutamine pathway to support redox maintenance, as well as an increase in glycolysis to divert nutrients into the glycosylation and pentose phosphate pathways. KRAS-driven cancers also recycle metabolites through increased autophagy or macropinocytosis to meet their increased demand for metabolites. Importantly, these KRAS-linked metabolic reprograming features differ according to KRAS copy number, tumor types, and genetic context [10,11]. In accordance, scientists pinpointed the development of biomarkers as critical to allow selection among patients bearing oncogene-driven tumors of those more likely to benefit from metabolic-targeting drugs [12].

In this study, we show that romidepsin-induced hyperacetylation caused depletion of stores of acetyl-CoA, the cell donor substrate for acetylation, and leads to lethal metabolic stress in KRAS-activated cells but not in KRAS-WT. We also confirmed that two acetyl-CoA metabolism pathways are hallmarks of sensitivity in a cell panel and in patients treated with an HDACi, validating not only these drugs as metabolism-targeting drugs, but also these pathways as a potential tool to identify a RAS-linked metabolic phenotype in a personalized medicine strategy.

## 2. Materials and Methods

### 2.1. Cell Lines, Treatment and Statistical Tests

The complete NCI-60 panel was obtained from National Cancer Institute (NCI) Anticancer Drug Screen (Bethesda, MD, USA). Pancreatic cell lines were obtained from ATCC (Manassas, VA, USA). All cells were cultivated in RPMI including 2 mM glutamine and 11.11 mM glucose supplemented by 10% FBS and penicillin/streptomycin (GIBCO, Grand Island, NY, USA).

Romidepsin (depsipeptide, NSC 630176) was provided by the Cancer Therapy Evaluation Program, NCI. Tariquidar was provided by the NCI Anticancer Drug Screen. PD-0325901 (MEK inhibitor), MK-2206 (AKT inhibitor) were purchased from ChemieTek (Indianapolis, IN, USA). Glutaminase inhibitor 968 compound was obtained from Millipore (Burlington, MA, USA); oligomycinA, NAC, glutathione, dimethyl-α-ketoglutarate, dimethyl-glutamate, sodium citrate, and sodium acetate were obtained from Sigma-Aldrich (St. Louis, MO, USA); DPI, ML171, and etomoxir was obtained from Selleckchem (Houston, TX). MitoQ was provided by Dan L. Sackett (NIH, NICHD). 

Unless otherwise specified, *t*-tests were performed to evaluate significance of results, and asterisks were added to graphs when the *p*-value was lower than 0.05. 

### 2.2. Transcriptomic Analysis with R

Gene expression basal levels for the NCI-60 and CCLE (Cancer Cell Line Encyclopedia) panels were downloaded via the cBio Cancer Genomics Portal [13]. Gene expression for acute myeloid leukemia samples was downloaded from the Gene Expression Omnibus (dataset GSE39363). Response to eight HDACi for CCLE panel were obtained from the Genomics of Drug Sensitivity in Cancer Project public database [14]. Gene expression for romidepsin-treated cells was obtained as follows. Cells were harvested after treatment and RNA extraction was performed using RNeasy Mini Kit (QIAGEN, Hilden, Germany), following the manufacturer’s instruction. Samples in triplicate were sent to the Frederick Cancer Research Core (NIH, NCI), where mRNA levels were determined by using Affymetrix GeneChip^®^ Human Genome U133 Plus 2.0 Array. Microarray data have been deposited in the NCBI Gene Expression Omnibus (accession number GSE133120). Detailed methods are provided in related figure legends.

Gene expression signature scores were calculated using weighted average expression (expression sum of down-regulated genes (*t*-test *t* < 0) subtracted from the expression sum of up-regulated genes (*t*-test *t* > 0) and divided by the total number of up- and down- regulated genes).

### 2.3. Mouse Study

Animal procedures were approved by the Animal Care and Use Committees at NIH/NIDDK. Athymic female mice (Ncr-nu/nu, 5–6 weeks old, ~20 g) were purchased from the Jackson Laboratory (JAX, Bar Harbor, ME, USA). Tumors were generated by injecting 10 million cells subcutaneously into the left flank. Treatment started once the tumor size reached an average of 100 mm^3^. Mice received 3 mg/kg romidepsin by intraperitoneal injection every 4 days and/or 5 mg/kg PD-0325901 by oral gavage every 4 days. Procedures are detailed in Supplementary Figure S1.

### 2.4. Flow Cytometry

Cell death was measured by flow cytometry after annexin V-FITC/ propidium iodide staining, according to manufacturer’s instructions (eBioscience, Inc., San Diego, CA, USA). Cell lines with a high expression of the multidrug efflux transporter P-glycoprotein (P-gp) according to the cellMiner database [15] were co-treated with a Pgp inhibitor to avoid the bias of drug efflux during the assessment of toxicity. Reactive Oxygen Species (ROS) quantification was performed with 10 µM H2DCF-DA (Abcam, Cambridge, UK) or 1 µM MitoSOX™ Red (Invitrogen, Carlsbad, CA, USA). In total, 1 µM tariquidar and 50 µM MK571 (Selleckchem) were added to the medium to inhibit fluorescent compound efflux by ABC transporters. The mitochondrial membrane potential was measured after incubation of cells in phenol red-free medium with 5 µM JC1 (Invitrogen) and 1 µM tariquidar. Cytochrome c release was measured by flow cytometry using the Cytochrome c Release Apoptosis Assay Kit (Millipore).

### 2.5. Western Blot

A western blot was performed as previously described [3]. Histone H3 antibodies were purchased from Cell Signaling Technology (Danvers, MA, USA), acetyl-histone H3 from Upstate Biotechnology (Lake Placid, NY, USA), γH2AX from Millipore, and GAPDH from American Research Products (Waltham, MA, USA). Original images of WB are presented in Supplementary Material.

### 2.6. Seahorse Experiment

Oxygen consumption rates (OCR) and extracellular acidification rates (ECAR) were measured using the XF96 or the XFp Extracellular Flux analyzer (Seahorse Bioscience, North Billerica, MA, USA). The protein amount per well was quantified using the BCA assay.

### 2.7. Cell Lines, Treatment and Statistical Tests

Metabolic profiling of A549 and ACHN cell lines was performed by Metabolon Inc. (Durham, NC, USA). After treatment, cells were pelleted, frozen, and sent for analysis (quadruplicates). All samples were randomly distributed across a single day platform run on GC/MS and LC/MS/MS instrumentation, and they were analyzed using Welch’s two-sample *t*-test.

### 2.8. Acetyl-CoA Quantification Analysis

Cells were harvested and sent frozen to the Metabolomics Core at the Translational Research Institute for Metabolism and Diabetes at Florida Hospital for estimation of acetyl-CoA and malonyl-CoA by LC-MS/MS.

## 3. Results

### 3.1. Activated RAS Pathway Is Linked to Romidepsin Sensitivity

**Figure 1:**
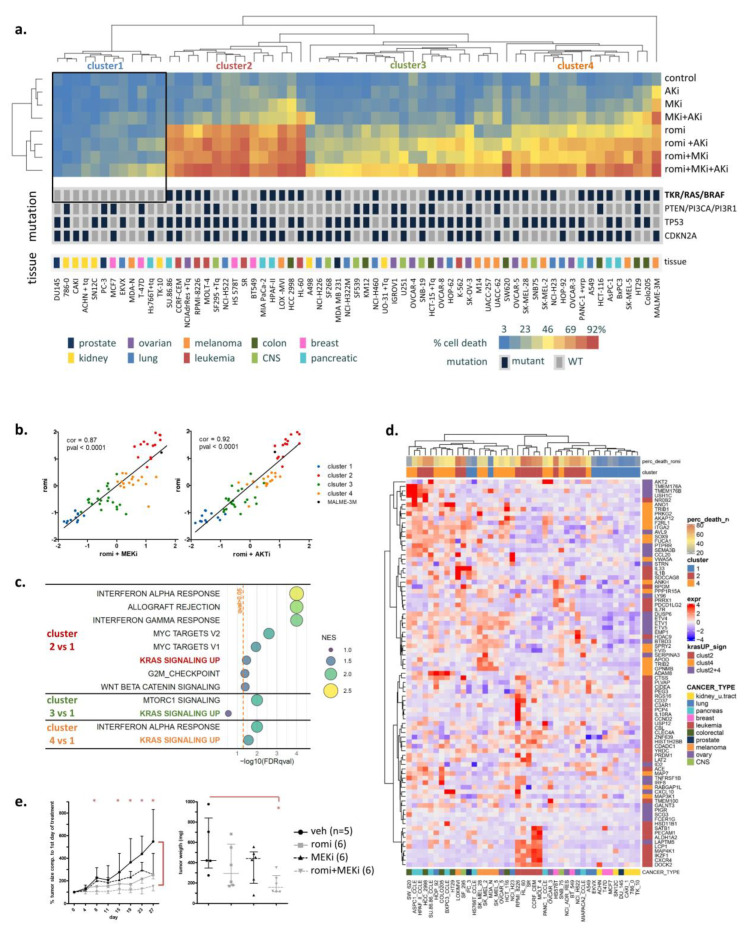
Activated RAS pathway is linked to romidepsin sensitivity. (**a**) Cells were treated with romidepsin (clinical dosing) and/or MEK inhibitor (MEKi, 250 nM) and/or AKT inhibitor (AKTi, 1 µM), and cell death was assessed at 48 h by annexin V-PI assay. Cell lines with a high expression of the multidrug efflux transporter P-glycoprotein were cotreated with P-gp inhibitor Tariquidar (Tq) to avoid the bias of drug efflux during the assessment of toxicity. Cells were hierarchically clustered according to percentage cell death, and a heatmap was drawn accordingly. Key DNA mutations and tissue origin of cancer are indicated below the heatmap. (**b**) The response to romidepsin in combination with either MEKi (**left**) or AKTi (**right**) for the 65 cell lines were plotted according to response to romidepsin alone (z-score). Cells were colored according to the four clusters obtained in (**a**). (**c**) SAM analysis was performed on NCI-60 panel gene expression between low responsive cluster (cluster 1) and each of the three others. SAM-ranked genes were analyzed with GSEA, and the significant enriched gene sets (FDR q-value < 0.05) obtained for each cluster was plotted according to normalized enrichment score (NES) and false discovery rate (FDR). (**d**). Leading-edge genes for “KRAS signaling up” signature obtained by GSEA analysis in clusters 2 and 4 were hierarchically clustered according to their basal expression in the 65 cell lines. (**e**) Athymic mice were injected with KRAS mutation bearing A549 cells forming tumor, then treated with romidepsin and/or MEKi for 28 days. Tumor size (median ± IQR) and mice weight (Supplementary Figure S2c) were measured over time, and tumor weight (median ± IQR) was measured after mice sacrifice and tumor dissection. Statistical difference between control and treated groups was determined using Welch’s *t*-test (unequal variance *t*-test), with * indicating a *p*-value < 0.05.

To identify the determinants of sensitivity to romidepsin alone or in combination with RAS downstream inhibitors (MEK and AKT inhibitors), we screened the NCI-60 panel (58 human cell lines from seven cancer tumor types), adding seven pancreatic cancer cell lines because they have a high prevalence of RAS mutation (97%) [1]. For this and all the experiments in this article, romidepsin was used with a dosing mimicking its concentration measured in the clinic, referred to as the “clinical dosing” (46 nM romidepsin, with drug removal at 6 h when the treatment time was over 6 h [16]). All the other treatments and co-treatments were added continuously for the indicated concentrations. The 65 cells were hierarchically clustered according to their response to treatment (Figure 1a), with four major clusters formed, mainly based on the response to romidepsin alone. The addition of RAS downstream inhibitors increased romidepsin efficacy proportionally (Figure 1b), with a 0.87 correlation in the 65 cell lines (*p*-value > 0.0001) between romidepsin alone and romidepsin in combination with the MEK inhibitor (MEKi, PD-0325901), and a 0.92 correlation (*p*-value > 0.0001) for the same analysis with the AKT inhibitor (AKTi, MK-2206). All but two of the 65 lines were insensitive to AKTi or MEKi alone. These results indicate that RAS downstream inhibitors did not trigger cell death, but amplified romidepsin-induced cell death. We queried mutations in well-known key cancer genes carried by these cell lines from the Cosmic Cancer Database. Eleven relevant genes were grouped in four categories with notable mutation: tyrosine kinase receptor (TKR)/RAS/BRAF pathway (EGFR, ERBB2, KRAS, NRAS, HRAS and BRAF), PI3K pathway (PTEN, PI3CA, and PI3R1), CDKN2A, and p53 (Figure 1a). Noticeably, the cluster of cells that did not respond to treatment correlated with the absence of mutation in the TKR/RAS/BRAF pathway, with a 0.47 correlation (*p*-value = 0.002). Cell doubling time, mutations in the PI3K pathway, CDKN2A, or p53 did not correlate with treatment efficacy (Supplementary Figure S1a,b). Basal expression levels for the 65-cell panel [13] were analyzed by a Significance Analysis of Microarrays (SAM) to identify significant genes in romidepsin non-sensitive and sensitive clusters. The significant genes were analyzed by Gene Set Enrichment Analysis (GSEA) software using the hallmark gene sets. Confirming the mutation analysis, “KRAS signaling up” gene signature was significantly enriched (nominal *p*-value < 0.01 and FDR q-value < 0.05) in the two romidepsin-sensitive cell clusters (Figure 1c). Gene expression value of the leading-edge genes from the signature in clusters 2 and 4 were plotted on a heatmap (Figure 1d). Again, cell lines resistant to romidepsin clustered together based on decreased “KRAS signaling up” signature expression. The heatmap also illustrated the variability in KRAS signature-related genes according to organ site. Together, these results showed an association between RTK/RAS/BRAF pathway activation and romidepsin sensitivity.

To assess the efficacy of the combination drug therapy in a preclinical model, we injected A549 cells (KRAS-mutant, cluster 4) subcutaneously in athymic mice and treated them with romidepsin and/or MEKi (Figure 1e and Figure S1c). From 15 days of treatment, the tumors treated with romidepsin/MEKi were significantly smaller than the untreated. Tumor weight at sacrifice was significantly lower in mice treated with the combination compared to untreated mice whereas no significant difference was noticed after single agent treatment, confirming the efficacy of the combination in vivo.

### 3.2. Early Cytoplasmic ROS Release Differentiates KRAS Sensitivity to Romidepsin, While Acetylation and DNA Damage Do Not

To understand why RTK/RAS/BRAF mutated cells were sensitive to romidepsin, we focused on the KRAS-mutant cells as a model. Progression in signaling events during romidepsin treatment using clinical dosing was evaluated in at least three KRAS-mutant and three KRAS-WT cell lines. 

**Figure 2:**
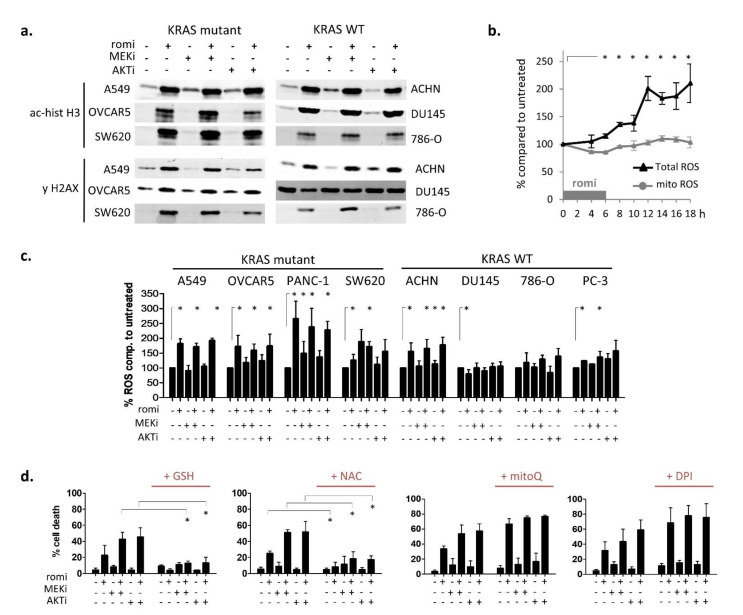
Early cytoplasmic ROS release differentiates KRAS sensitivity to romidepsin, while acetylation and DNA damage do not. (**a**) Histone acetylation and H2AX phosphorylation (DNA damage marker) were measured by WB in cells treated for 6 h with romidepsin ± MEKi or AKTi. (**b**) Total and mitochondrial ROS production were measured over time by flow cytometry for 18 h during treatment with romidepsin (clinical dosing) in A549 cell line. (**c**) Total ROS production was measured at 24 h in cell lines after romidepsin (clinical dosing) ± MEKi ± AKTi. (**d**) Percentage cell death was measured at 48 h in A549 cell line after treatment with romidepsin ± MEKi ± AKTi, in presence or not of cytoplasmic ROS scavengers (5 mM GSH or NAC), mitochondrial ROS scavenger (1 µM MitoQ), or pan-NADPH oxidase inhibitor (5 µM DPI). In (**b**–**d**), data are plotted as mean of three experiments ± SD, and statistical significance between groups was assessed using unpaired two-tailed Student’s *t*-test (* indicates a *p*-value < 0.05).

Histone acetylation, the main target of romidepsin, and DNA damage (using the commonly accepted marker γH2AX) were measured by western blot after 6 h combination treatment in six cell lines (Figure 2a). No differences were observed between KRAS-mutant and KRAS-WT cells. Both sensitive and resistant cells undergo increased histone acetylation when treated with romidepsin, and five out of six cell lines had increased DNA damage. MEKi and AKTi did not increase these signals.

Next, total and mitochondrial reactive oxygen species (ROS) production were measured over time in the A549 cell line treated with romidepsin by flow cytometry. Total ROS increased approximately 10–12 h, whereas no increase was detected in mitochondrial ROS over 18 h (Figure 2b). Total ROS production was then evaluated in eight cell lines after a 24 h combination treatment (Figure 2c). ROS production was mainly induced in romidepsin-sensitive KRAS-mutant cells: only one out of four KRAS-WT cells had a significant romidepsin-induced ROS increase, whereas all four KRAS-mutant cells showed an increase. MEKi and AKTi did not increase ROS signaling. 

ROS involvement in cell death at 48 h was evaluated after the 6 h romidepsin treatment. Scavenging of total ROS by glutathione (GSH) or *N*-acetyl-cysteine (NAC) significantly rescued all KRAS-mutant cells from death, whereas neither mitochondrial ROS scavenger (mitoQ) nor NADPH oxidase (DPI) inhibitor did (Figure 2d and Figure S2a). We also assessed mitochondrial ROS production, mitochondrial membrane depolarization and the apoptotic marker cytochrome c release at 24 h and 48 h (Supplementary Figure S2b). These signals—indicating the onset of cell death—were low at 24 h but high at 48 h in romidepsin-treated KRAS-mutant cells. A combination with MEKi or AKTi amplified most of these late death signals at 48 h.

Taken together, these results indicated that romidepsin induces early cytoplasmic ROS production in KRAS-mutant cells. Mitochondrial ROS production and mitochondrial membrane depolarization appeared late, suggesting that they were apoptotic by-products rather than drivers of apoptosis. Having eliminated the main known causes of ROS production (NADPH oxidase and mitochondria), we therefore explored other possible sources of cytoplasmic ROS imbalance after romidepsin treatment. 

### 3.3. Romidepsin Affects Two TCA-Fueling Pathways: Fatty Acid Beta-Oxidation and Glutaminolysis

**Figure 3:**
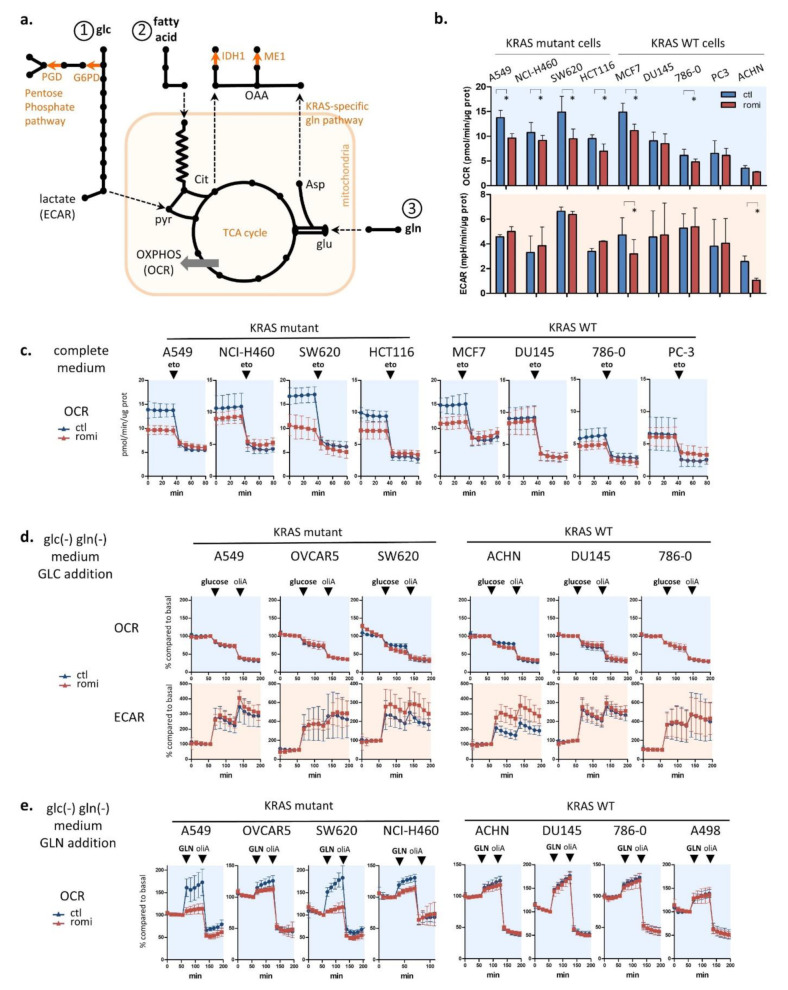
Romidepsin affects two TCA-fueling pathways: fatty acid beta-oxidation and glutaminolysis. (**a**) Schematic view of cytoplasmic ROS scavenging through NADPH producing-metabolic enzymes and their link with the three main TCA cycle fueling pathways (1/glycolysis, 2/beta-oxidation, 3/glutaminolysis). Cytoplasmic NADPH producing enzymes are downstream of glutaminolysis in KRAS-mutant cells (ME1), downstream of TCA cycle through citrate production (IDH1), or downstream of the first step of glycolysis (PGD and G6PD in pentose phosphate pathway). (**b**) Basal OXPHOS and glycolysis were evaluated by measuring oxygen consumption rate (OCR) and extracellular acidification rate (ECAR) using a Seahorse XF96 in four KRAS-mutant and five KRAS-WT cell lines after 18 h treatment romidepsin (clinical dosing). (**c**) The involvement of fatty acid β-oxidation in TCA cycle fueling was evaluated by adding 40 µM etomoxir to cell lines while OCR was measured in four KRAS-mutant and four KRAS-WT cell lines treated for 18 h with romidepsin. (**d**) The involvement of glycolysis in TCA cycle fueling was evaluated by adding 10 mM glucose to glucose/glutamine-free medium while OCR and ECAR were measured in three KRAS-mutant and three KRAS-WT cell lines treated for 18 h with romidepsin. (**e**) The involvement of glutaminolysis in TCA cycle fueling was evaluated by adding 2 mM glutamine to glucose/glutamine-free medium while OCR was measured in four KRAS-mutant and four KRAS-WT cell lines treated for 18 h with romidepsin. In (**b**–**e**), data were plotted as mean ± SD, and statistical significance between groups was assessed using an unpaired two-tailed Student’s *t*-test in (**b**), with * indicating a *p*-value < 0.05. To assess the role of glycolysis, cells were glucose- and glutamine-deprived for 1 h, and then glucose was added to the medium while OCR and ECAR were measured. Glucose addition increased glycolysis (ECAR) in all six tested cell lines, pretreated or not with romidepsin (**d**). In contrast, OCR decreased after glucose addition, reflecting a shift from OXPHOS to glycolysis as an immediate source of ATP. The subsequent addition of oligomycin A, which blocks ATP-linked respiration and forces cells to rely on glycolysis to produce ATP, allowed measurement of maximum glycolytic capacity, also unchanged after the HDACi treatment.

Four enzymes in metabolic pathways produce cytoplasmic NADPH, an intrinsic ROS scavenger that maintains a cytoplasmic redox balance (Figure 3a). We hypothesized that romidepsin may affect metabolic pathways that regulate NADPH production and therefore ROS sensitivity. To investigate, we explored the metabolic effect of romidepsin by measuring in real time cell oxygen consumption rates (OCR), a surrogate of oxidative phosphorylation (OXPHOS), and cell extracellular acidification rates (ECAR), a surrogate of glycolysis. We first assessed basal OCR and ECAR in nine cell lines treated for 18 h with romidepsin using a clinical dosing (Figure 3b). Romidepsin treatment decreased OXPHOS in the four KRAS-mutant cells and in two out of five KRAS-WT cells. Next, by varying fatty acid, glucose or glutamine supply we could evaluate the three main tricarboxylic acid (TCA) cycle fueling pathways: fatty acid beta-oxidation, glycolysis, and glutaminolysis. We evaluated fatty acid β-oxidation-supported OXPHOS by adding etomoxir, an inhibitor of carnitine palmitoyltransferase-1, the rate-limiting-enzyme in β-oxidation. Etomoxir decreased OCR for all untreated cell lines, indicating that all cell lines relied on β-oxidation to support OXPHOS (Figure 3c). The etomoxir effect on romidepsin-treated cells was significantly reduced compared with untreated cells for seven out of eight cell lines, indicating that romidepsin inhibits β-oxidation-supported OXPHOS (Figure 3c and Figure S3). 

To assay whether glutamine was a dominant OXPHOS fuel source rather than glucose, the same experiment was performed in 1 h-glucose/glutamine-deprived cells, and glutamine was subsequently added (Figure 3e). In all untreated cells, glutamine addition caused an increase in OCR, indicating that all cells used glutamine for OXPHOS. More importantly, in three out of four KRAS-mutant cell lines, romidepsin inhibited the ability of the cells to shift to OXPHOS after the addition of glutamine. This effect was not observed in the four KRAS-WT cell lines. 

Together, these results indicated that cancer cell lines used β-oxidation and glutamine rather than glucose as the main source of fuel for the TCA cycle, independently of KRAS status. Romidepsin impaired OXPHOS via inhibition of β-oxidation in almost all tested cell lines, but impaired glutamine use for OXPHOS only in cells bearing KRAS mutations.

### 3.4. Romidepsin Affects Acetyl-CoA Availability

**Figure 4:**
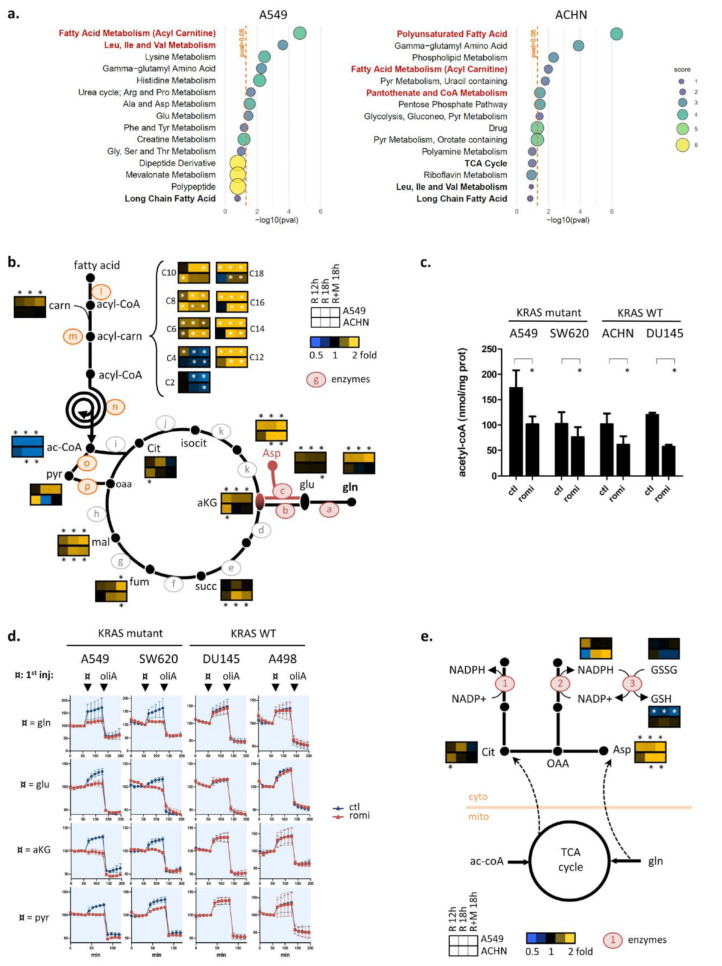
Romidepsin affects acetyl-CoA availability (**a**) 620 metabolites were quantified in A549 and ACHN cell lines after 18 h romidepsin treatment (clinical dosing), and the top 15 enriched metabolic pathways were obtained after enrichment pathway analysis on significantly modified compounds (Welsh’s *t*-test). *p*-values for pathway scores were calculated using hypergeometric test. (**b**) 620 metabolites were quantified after treatment with romidepsin (clinical dosing) for 12 h, 18 h and 18 h in combination with MEKi, and those involved in β-oxidation, glutaminolysis and TCA cycle were overlaid on TCA cycle fueling pathway representation (median fold change). Letters refer to enzymes whom expression was quantified by microarray analysis, and for which results are presented in Supplementary Figure S5c. Statistical difference between control and treated groups was evaluated by Welsh’s *t*-test (**c**) Acetyl-CoA levels were quantified by mass spectrometry in four cell lines following 18 h romidepsin treatment. Data are plotted as mean ± SD, and statistical difference was assessed using an unpaired two-tailed Student’s *t*-test. (**d**). Cells were treated for 18 h with romidepsin, then incubated for 1 h in medium without glucose and glutamine, and then variation in OXPHOS (OCR) was measured by Seahorse analysis while either glutamine, cell permeant-glutamate, cell permeant-αKG or pyruvate was subsequently added, followed by oligomycin A. (**e**) Metabolites involved in cytoplasmic redox balance downstream of the TCA cycle are represented, including the GSH recycling step requiring NADPH to reconvert GSSG (oxidized glutathione) to GSH (reduced glutathione) (see (**b**) for detailed legend information). Numbers refer to enzymes for which expression levels are presented in Supplementary Figure S4e. In (**b**,**c**,**e**), * indicates a *p*-value < 0.05.

To understand the disturbances in β-oxidation and glutamine metabolism induced by romidepsin, a metabolomic analysis was performed in A549 (KRAS-mutant) and ACHN (WT) cell lines. Cells were treated 12 h or 18 h with romidepsin using clinical dosing, and 629 metabolites were quantified. Romidepsin-altered pathways were determined by Metabolync pathway enrichment analysis. Fatty acid metabolism and TCA cycle pathways were significantly modified in both cell lines at 12 h and/or 18 h (Figure 4a and Figure S4a), as well as valine/leucine/isoleucine metabolism, another source of the TCA cycle-fueling metabolite acetyl-CoA, confirming OCR/ECAR data (Figure 3).

Metabolites in the TCA cycle and in its two fueling pathways (glutaminolysis and β-oxidation) are reported in Figure 4b, and metabolites from valine/leucine/isoleucine degradation pathway and glycolysis in Supplementary Figure S4b,c. The TCA cycle metabolite malate accumulated in both cell lines after 18 h, indicating a block in TCA cycle flux. In the β-oxidation pathway, C6- to C18- acyl-carnitine (upstream) accumulated while acetyl-CoA (downstream) was strongly depleted in both cell lines. These data suggest that romidepsin induced a block in fatty acid flux toward the TCA cycle, similar to what was observed with OCR/ECAR experiments (Figure 3). Glutamine, glutamate, and αKG accumulated only in KRAS-mutant cells, confirming the decreased ability of these cells to use glutamine for TCA cycle following romidepsin treatment. 

Notably, among all the quantified metabolites in the TCA fueling pathway, only acetyl-CoA and its two direct precursors downstream of β-oxidation (C2- and C4-acylcarnitine) were depleted, whereas almost all other TCA-fueling metabolites were accumulated (Figure 4b). To validate the metabolomics analysis, we quantified cellular acetyl-CoA in four cell lines by mass spectrometry. Romidepsin induced a decrease in acetyl-CoA in the two KRAS-mutant cell lines as well as in the two KRAS-WT ones (Figure 4c). Knowing that the rate of flow through TCA is limited by the availability of its substrates oxaloacetate and acetyl-CoA, these data suggest that acetyl-CoA depletion caused a block in TCA cycle metabolite flux in KRAS-mutant cells, consequently causing an accumulation of glutamine downstream metabolites that were feeding into the cycle in the KRAS cells only. 

To explore this hypothesis, we returned to the Seahorse experiments. Cells were placed for 1 h in glucose/glutamine-deprived medium, then glutamine, DM-glutamate (cell permeable glutamate), DM-glutarate (cell permeable αKG) or pyruvate were added while OCR was measured (Figure 4d). After 18 h romidepsin treatment, KRAS-mutant cells could not use glutamine, glutamate, αKG, or pyruvate for OXPHOS, whereas no variation in the use of these metabolites was observed between treated and untreated cells in KRAS-WT cells. The fact that αKG was not used for OXPHOS in romidepsin-treated KRAS-mutant cells indicated that the decrease in glutamine flux induced by romidepsin was not due to decreased activity in glutaminolysis enzymes (GLS, GOT2, and GLUD1). It indicated an event downstream of the αKG formation step. Romidepsin also inhibited cellular ability to use pyruvate in the KRAS-mutant cells. These findings confirmed that romidepsin induced a block in TCA cycle flux downstream of glycolysis and glutaminolysis, leading to accumulation of unused fueling metabolites (seen in Figure 4b), and a decrease of OXPHOS in KRAS-mutant cells (seen in Figure 3b). 

When looking at the cytoplasmic NADPH-producing pathways downstream of TCA cycle and/or glutaminolysis (described in Figure 3a), we observed that the reduced form of GSH, the end product of NADPH-producing pathways and the main cell defense against ROS, was also significantly reduced in the A549 cell line but not in ACHN (Figure 4e). 

Taken together, we postulated that that romidepsin-induced acetyl-CoA depletion was the cause of TCA cycle flux inhibition, which itself led to a decreased GSH recycling.

### 3.5. Acetyl-CoA Precursors Replenish Acetyl-CoA Stock and Rescue KRAS-Mutant Cells from Metabolic Stress

Acetyl-CoA is a central metabolite that links cell energy status and transcription regulation. It is a TCA cycle limiting factor, as well as the only substrate for histone acetylation, the epigenetic mechanism regulating transcription [17]. To gain further insight into romidepsin-modified acetyl-CoA metabolism, microarray analysis was performed on A549, MIA PaCa-2, and ACHN cell lines after 6 h or 18 h romidepsin treatment (clinical dosing) and pathway enrichment analysis using the KEGG signature database to more thoroughly cover modifications in metabolic pathways. Pathway mining highlighted modifications in fatty acid metabolism, valine/leucine/isoleucine degradation, and steroid biosynthesis in the top 10 modified pathways for the three tested cell lines (Supplementary Figure S5a). Nevertheless, even if these pathways are involved in acetyl-CoA metabolism, variation in the expression of acetyl-CoA metabolism genes was not involved in acetyl-CoA depletion, and even trended to counter it with increasing levels of enzymes producing acetyl-CoA and decreasing levels of enzymes using acetyl-CoA (Supplementary Figure S5b,c). 

Since modification in metabolic enzyme mRNA level was not the cause of acetyl-CoA depletion, we looked at other possible reasons. Noticeably, acetyl-CoA is the sole acetyl-group donor for protein acetylation. Yet, romidepsin is a histone deacetylase inhibitor, so it causes abnormally high cell acetylation levels and thus an abnormally high acetyl group consumption that could be the main cause of acetyl-CoA depletion. We therefore assessed whether restoring acetyl-CoA metabolite levels could be sufficient to rescue the cell damages that we observed after romidepsin treatment. 

**Figure 5:**
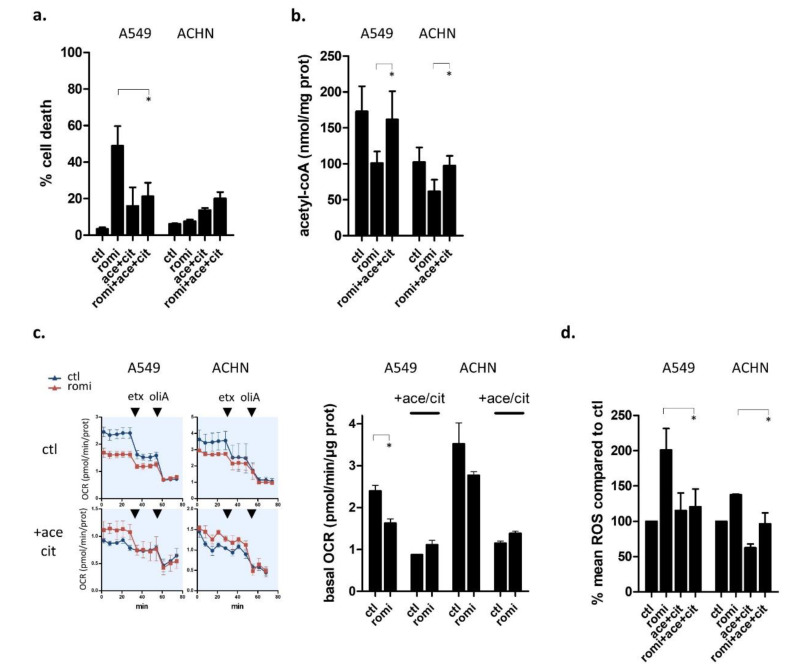
Acetyl-CoA precursors replenish acetyl-CoA stock and rescue KRAS-mutant cells from metabolic stress. (**a**) Cell death was measured by flow cytometry (annexin-V positive cells) in cells treated or untreated for 48 h with romidepsin (clinical dosing), in the presence or absence of 20 mM acetate + 5 mM citrate. (**b**) Acetyl-CoA was quantified by mass spectrometry in KRAS-mutant (A549) and KRAS-WT (ACHN) cell lines treated or untreated with romidepsin (18 h), in presence or absence of 20 mM acetate + 5 mM citrate. (**c**) OXPHOS (OCR) was measured in cells treated or untreated for 18 h with romidepsin, in presence or absence of 20 mM acetate + 5 mM citrate followed by addition of 40 µM etomoxir then 5 µM oligomycin A (**left**). Basal OCR level for romidepsin-treated and untreated cells was calculated and plotted according to presence or absence of acetyl-CoA precursors in medium (**right**). (**d**) ROS production was measured by flow cytometry (DCF-DA) in cells treated or untreated for 24 h with romidepsin, in presence or absence of 20 mM acetate + 5 mM citrate. For all graphs, data are plotted as mean ± SD, and statistical difference was assessed using an unpaired two-tailed Student’s *t*-test (* indicates a *p*-value < 0.05).

To reintroduce acetyl-CoA in cells, we incubated cells with two cytoplasmic acetyl-CoA precursors (citrate and acetate), because acetyl-CoA itself cannot cross the cytoplasmic membrane. Various precursor concentrations were tested on three KRAS-mutant cell lines and we selected the optimal conditions for cell death rescue (Supplementary Figure S6). Addition of 5 mM acetate plus 20 mM citrate in romidepsin-treated A549 cell line rescued the KRAS-mutant cells from romidepsin-induced cell death (Figure 5a). We then verified that citrate/acetate did replenish acetyl-CoA stock at this concentration. Mass spectrometry quantification of acetyl-CoA level in A549 and ACHN confirmed that addition of acetate/citrate in romidepsin-treated cells replenished acetyl-CoA stock with levels equivalent to those observed in untreated cells (Figure 5b). These results also indicated that the acetyl-CoA producing enzymes ACLY (catalyzing citrate to acetyl-CoA), ACSS1 (catalyzing acetate to mitochondrial acetyl-CoA), and ACSS2 (catalyzing acetate to cytoplasmic acetyl-CoA) were not themselves affected by romidepsin treatment, neither in terms of transcription (confirming Supplementary Figure S5 results), translation, or post-translation.

We then investigated the coupling between acetyl-CoA depletion and cell death. In the presence of acetyl-CoA precursors, romidepsin did not induce a decrease in basal OCR levels in the KRAS-mutant cells, contrary to what was observed in the absence of citrate/acetate (Figure 5c). We also measured romidepsin-induced cytoplasmic ROS production in presence or not of acetyl-CoA precursors, and we observed that acetyl-CoA replenishment inhibited romidepsin-induced oxidative stress in KRAS-mutant cells (Figure 5d). 

These results showed that the decrease in acetyl-CoA levels was a leading cause of romidepsin-induced cell death in KRAS-mutant cells. Romidepsin-induced acetyl-CoA decrease caused a reduction in TCA cycle flux and an increase in cytoplasmic ROS production that triggered cell death in KRAS-mutant cells only. Notably, romidepsin-induced damage was reversed by just replenishing acetyl-CoA stocks, and it was not caused by romidepsin-induced inhibition of acetyl-CoA generation pathways, suggesting that it was hyperacetylation-induced acetyl-CoA overconsumption that led to the decrease in acetyl-CoA.

### 3.6. Two Acetyl-CoA Metabolism Pathways Are Markers of HDAC Inhibitor Sensitivity

Because romidepsin-induced acetyl-CoA decrease was observed in all tested cell lines, whereas only KRAS-mutant cell lines showed a decrease in acetyl-CoA-linked cell death after HDAC inhibition, we hypothesized that KRAS-mutant cells were more acetyl-CoA-dependent than KRAS-WT cell lines. 

**Figure 6:**
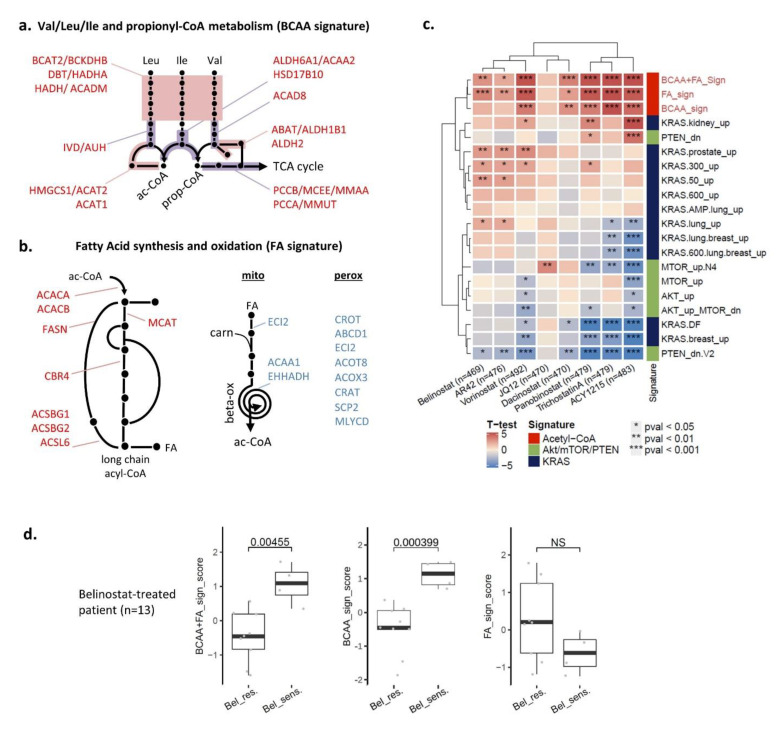
Two acetyl-CoA metabolism pathways are markers of HDAC inhibitor sensitivity. (**a**,**b**) Key acetyl-CoA metabolic pathways involved in response to eight HDAC inhibitors were selected by elastic net analysis in CCLE panel (see Supplementary Figure S7). Leading-edge genes for the acetyl-CoA pathways were overlaid onto pathway representations, with upregulated genes in red and downregulated genes in blue. (**c**) Three gene signatures were derived from the two pathways and signature scores were calculated in CCLE panel by weighted averaged expression using both up- and down-regulated genes. KRAS activation signatures were also calculated by the same method using the C6 collection in the Molecular Signatures Database. Significant differences for all signature scores between resistant versus sensitive cell lines were calculated by Welch’s *t*-test in CCLE panel for eight HDACis, and results were plotted in a heatmap. Numbers in parentheses indicate the number of cell lines tested for each HDACi. (**d**) Acetyl-CoA Signature scores were plotted (boxplot with individual data point) according to response to Belinostat in AML patients (independent cohort). Statistical significance between groups was assessed using unpaired two-tailed Student’s *t*-test (NS stands for “not significant”).

To determine whether HDACi-sensitive cells had an acetyl-CoA altered metabolism at baseline, we derived an acetyl-CoA-related signature from transcriptomic datasets. We used the CCLE panel (608 cell lines) with a known response to eight HDACis to gather enough samples to gain a sufficient statistical power [14]. A machine learning approach was taken to identify key metabolic pathways related to HDACi sensitivity and to select the acetyl-CoA-related ones. The pipeline is illustrated in Supplementary Figure S7. Among the five retained pathways, two were involved in branched chain amino acid (BCAA) metabolism (valine/leucine/isoleucine degradation and propionyl-CoA catabolism), while three were involved in fatty acid (FA) metabolism (FA biosynthesis, mitochondrial FA β-oxidation, and peroxisomal FA β-oxidation). Leading-edge genes for the five pathways were obtained by GSEA analysis in the CCLE panel and overlaid onto pathway representations with gene expression direction in Figure 6a,b. Three gene expression signatures (GES) were derived from the leading-edge genes: BCAA signature, FA signature, and a combination of BCAA and FA signatures. Association of the three GES with the eight HDACi was analyzed in the training cohort (CCLE panel) and compared with several RAS activation signature pathways (and Akt/mTOR as negative control) derived from the GSEA Molecular Signatures Database (Figure 6c). For the BCAA+FA and BCAA signatures, increased scores were significantly linked with sensitivity to seven of eight HDACi, and FA signature to five of eight HDACi. Acetyl-CoA related signature were more informative than KRAS signaling ones, which showed association with a maximum of three HDACi, with *p*-values of less significance. 

To validate the importance of acetyl-CoA for HDACi sensitivity in clinical samples, we tested the performance of the three GES in samples obtained from 13 acute myeloid leukemia (AML) patients for whom microarray data prior to treatment and clinical response to belinostat are publicly available (Figure 6d). The combined BCAA+FA and BCAA signatures were both validated in this independent validation dataset, with *p*-value = 0.0045 and 0.0004, respectively, while the FA signature alone could not differentiate sensitive and resistant (*p* = 0.07). These results confirmed that samples with an altered expression profile for acetyl-CoA metabolism at baseline have the strongest sensitivity to HDACi.

Together, these results confirmed that alteration in two acetyl-CoA metabolism pathways (FA metabolism and BCAA metabolism) were a hallmark of sensitivity to HDACi in patients. It also validated the two acetyl-CoA derived GES as possible tools to further select patients for whom HDACi could be beneficial.

## 4. Discussion

In this study, we discovered that the class I HDAC inhibitor romidepsin caused a depletion in acetyl-CoA, most probably through an overconsumption of acetyl-CoA itself triggered by induced hyperacetylation of histones. We also uncovered a strategy for treating KRAS pathway modified tumors. Indeed, in RAS-activated cells, the depletion in acetyl-CoA triggered a decrease in TCA cycle flux combined with an increase in cytoplasmic ROS that led to cell death. Computational analysis of transcriptomic profiles in a 608-cell panel highlighted inherent alterations in acetyl-CoA generation pathways that could cause a higher dependency on acetyl-CoA depleting drugs, and we confirmed our results in samples from patients treated with belinostat. Together, these findings provide a promising strategy for improving treatments for the RAS pathway-modified tumors, where acetyl-CoA metabolism signatures could be exploited to select patients who are most likely to benefit from HDACi treatment.

The tight link between histone acetylation and acetyl-CoA levels is well known but has been described in only one direction: acetyl-CoA levels determine histone acetylation levels. In fact, the abundance of the nucleo-cytosolic pool has a direct impact on the enzymatic activity of HATs [18]. Here, we reported the other direction of this relationship: forcing cells to acetylate histones by HDAC inhibition leads to a depletion in acetyl-CoA.

This is consistent with the fact that acetyl-CoA is the unique donor of acetyl groups for acetylation, and that even after 6 h treatment followed by 12 h washout, the acetylated histone signal did not fade (Supplementary Figure S8), implying that sequestrating the “acetyl group” pool on histones can be a long-lasting event, preventing the quick restoration of acetyl-CoA level. In agreement, Kurdistani [19] hypothesized that acetylated histones might function as a storage site for acetate to be released by HDACs when the need arises. His theory supports our results implying that HDAC inhibition leads to an imbalance in acetyl-CoA pool by inhibiting its release.

In normal physiology, fluctuations in acetyl-CoA concentration reflect the metabolic state of the cell. Due to this key role, its regulation is tightly controlled by three different mechanisms: the expression of its metabolic enzymes or their regulators, its cellular and subcellular compartmentalization, and the post-translational regulation of the enzymes involved in its production (by acetylation) [17,18,20]. Several lines of evidence lead us to conclude that it is histone acetylation per se rather than any other mechanism that causes depletion of the acetyl pools. First, we evaluated transcription modulation (Figure 5). Second, we indirectly evaluated whether increased protein acetylation could have caused the metabolic defects. Admittedly, ACSS1, ACSS2, ACLY, and PDHA1 are involved in acetyl-CoA formation and their activity is inhibited by acetylation [20,21]. However, the addition of acetate plus citrate in romidepsin-treated cells restored acetyl-CoA levels to those observed in untreated cells (Figure 6a), indicating that these enzymes were not inhibited. Further, the malate and succinate dehydrogenase (MDH and SDH) enzymes in the TCA cycle are also regulated by acetylation, but OCR/ECAR experiments did not show inhibition of these enzymatic steps (Figure 4c).

Another point to discuss is the link between TCA cycle and cytoplasmic ROS production. Gansemer et al. [22] found that decreasing the availability of the TCA cycle substrate acetyl-coA led to a decrease in cytoplasmic reduced glutathione, much as we observed. As the authors stated in the discussion, it is still difficult to estimate the precise contribution of TCA cycle flux inhibition in reduced redox capacity. Other cytoplasmic pathways may also affect redox, such as pentose phosphate pathway (with G6PD and PGD), one-carbon folate metabolism (via MTHFD), and ALDH (involved in several pathways). In addition, redox-sensitive couples like GSSG/GSH and NADP+/NADPH are interconnected between compartments, and the disturbance in one compartment usually affects the whole cell [23]. While our results suggested a preponderance of TCA cycle perturbation and cytoplasmic ROS in romidepsin-induced cell death, these other pathways and compartments remain to be explored. Several teams have already highlighted the connection between HDACis and energetic metabolism. For example, OCR decrease was induced with pan-HDACis, like butyrate, panobinostat, or trichostatin A [24,25,26]. Others have demonstrated that HDACis perturbed fatty acid metabolism, with variation in intermediate metabolites very similar to the ones we observed in our metabolomics analysis (Figure 4b and [27,28,29,30,31]). Although others have shown that HDACis inhibit glycolysis [29,32], there was no significant variation in our hands. These differences could reflect the very important fact that the source of carbon used to fuel metabolism is context dependent. Several teams have indeed demonstrated that glutamine is the predominant carbon source for mitochondrial metabolism in vitro, whereas glucose contributes to a greater degree in vivo [11,33]. Confirming these analyses, in our in vitro experiments, cells did not use glucose for lactate production or for OXPHOS, except when they were glutamine-starved, or when beta-oxidation was inhibited. These differences between in vivo and in vitro experiments could be a barrier to a translation into clinical therapy. Nevertheless, romidepsin induced tumor growth inhibition in our mouse model, confirming the activity of romidepsin in a KRAS-mutated tumor model in which glucose is presumably preferred over glutamine. Also, the combination of panobinostat with β-oxidation inhibitor etomoxir synergistically reduced tumor growth compared with single-agent treatments in a glioblastoma PDX model [30], corroborating our results. Moreover, gene expression analysis from romidepsin-treated cutaneous T-cell lymphoma patients also revealed acetyl-CoA metabolism perturbation (Supplementary Figure S5c). Recently, pancreatic cancer has been shown to undergo reprogramming in lipid-related and acetyl-CoA metabolism pathways [34,35,36], extending our finding about the importance of acetyl-CoA metabolism in romidepsin sensitivity in KRAS-activated cell lines. Accordingly, a phase II study in patients with KRAS-mutant NSCLC is ongoing with the fatty acid synthase inhibitor TVB-2640 (NCT03808558). Preliminary data indicate pharmacodynamic effects and evidence of clinical activity for TVB-2640 in patients with tumors bearing KRAS mutations [37]. These preliminary clinical results are very encouraging in a field where only a handful of metabolism-targeting drugs have made it into clinical trials, despite the evidence of high tumor metabolic demand. 

## 5. Conclusions

In sum, our results suggest that a dominant effect of HDAC inhibition is the depletion of acetyl-CoA. While the canonical mechanism of opening chromatin to facilitate gene transcription is undoubtedly important in some settings, the rapid cell death in T-cell lymphoma cells is more consistent with a metabolic impact. One future direction will be to determine what role acetyl-CoA could play in the myriad other effects observed following HDAC inhibition, including R-loop persistence, kinetochore assembly and DNA damage.

Another direction will be to determine whether this metabolic vulnerability could be exploited in the clinic. Acetyl-CoA metabolism signatures were performant in a small validation cohort of patients, and will have to be tested in a prognostic study in order to confirm their strength for future clinical use as biomarkers to help stratifying patients that should receive HDACi. Our results also open doors to new combinations to treat patients with solid cancer using HDACi with FA or BCCA metabolism inhibitors. 

## Data Availability

The expression data are deposited in Gene Expression Omnibus (GEO; https://www.ncbi.nlm.nih.gov/geo/) (accessed on 25 May 2022) under the accession number GSE133120. Metabolomic data are available on request.

## Supplementary Materials

The following supporting information can be downloaded at: https://www.mdpi.com/article/10.3390/cancers14112643/s1, Figure S1: Additional information related to Figure 1; Figure S2: Additional information related to Figure 2; Figure S3: Additional information related to Figure 3; Figure S4: Additional information related to Figure 4; Figure S5: Variation in acetyl-CoA metabolism gene expression counters romidepsin-induced perturbation; Figure S6: Additional information related to Figure 5; Figure S7: Analysis of HDACi sensitivity in CCLE panel; Figure S8: Histone acetylation over time in cell lines treated with romidepsin (clinical dosing). Original images of WB.

## References

[1] Ryan M.B., Corcoran R.B. (2018). Therapeutic Strategies to Target RAS-Mutant Cancers. Nat. Rev. Clin. Oncol.

[2] Mattiuzzi C., Lippi G. (2019). Current Cancer Epidemiology. J. Epidemiol. Glob. Health.

[3] Bahr J.C., Robey R.W., Luchenko V., Basseville A., Chakraborty A.R., Kozlowski H., Pauly G.T., Patel P., Schneider J.P., Gottesman M.M. (2016). Blocking Downstream Signaling Pathways in the Context of HDAC Inhibition Promotes Apoptosis Preferentially in Cells Harboring Mutant Ras. Oncotarget.

[4] Hanker A.B., Healy K.D., Nichols J., Der C.J. (2009). Romidepsin Inhibits Ras-Dependent Growth Transformation of NIH 3T3 Fibroblasts and RIE-1 Epithelial Cells Independently of Ras Signaling Inhibition. J. Mol. Signal..

[5] Choudhary S., Wang H.-C.R. (2007). Proapoptotic Ability of Oncogenic H-Ras to Facilitate Apoptosis Induced by Histone Deacetylase Inhibitors in Human Cancer Cells. Mol. Cancer Ther..

[6] Bates S.E. (2020). Epigenetic Therapies for Cancer. N. Engl. J. Med..

[7] Safari M., Litman T., Robey R., Aguilera A., Chakraborty A.R., Reinhold W., Basseville A., Petrukhin L., Scotto L., O’Connor O.A. (2021). R-Loop-Mediated SsDNA Breaks Accumulate Following Short-Term Exposure to the HDAC Inhibitor Romidepsin. Mol. Cancer Res..

[8] King J., Patel M., Chandrasekaran S. (2021). Metabolism, HDACs, and HDAC Inhibitors: A Systems Biology Perspective. Metabolites.

[9] Kimmelman A.C. (2015). Metabolic Dependencies in RAS-Driven Cancers. Clin. Cancer Res..

[10] Kerr E.M., Gaude E., Turrell F.K., Frezza C., Martins C.P. (2016). Mutant *Kras* Copy Number Defines Metabolic Reprogramming and Therapeutic Susceptibilities. Nature.

[11] Hensley C.T., Faubert B., Yuan Q., Lev-Cohain N., Jin E., Kim J., Jiang L., Ko B., Skelton R., Loudat L. (2016). Metabolic Heterogeneity in Human Lung Tumors. Cell.

[12] Nussinov R., Jang H., Tsai C.-J., Cheng F. (2019). Review: Precision Medicine and Driver Mutations: Computational Methods, Functional Assays and Conformational Principles for Interpreting Cancer Drivers. PLoS Comput. Biol..

[13] Cerami E., Gao J., Dogrusoz U., Gross B.E., Sumer S.O., Aksoy B.A., Jacobsen A., Byrne C.J., Heuer M.L., Larsson E. (2012). The CBio Cancer Genomics Portal: An Open Platform for Exploring Multidimensional Cancer Genomics Data. Cancer Discov..

[14] Yang W., Soares J., Greninger P., Edelman E.J., Lightfoot H., Forbes S., Bindal N., Beare D., Smith J.A., Thompson I.R. (2013). Genomics of Drug Sensitivity in Cancer (GDSC): A Resource for Therapeutic Biomarker Discovery in Cancer Cells. Nucleic Acids Res..

[15] Reinhold W.C., Sunshine M., Liu H., Varma S., Kohn K.W., Morris J., Doroshow J., Pommier Y. (2012). CellMiner: A Web-Based Suite of Genomic and Pharmacologic Tools to Explore Transcript and Drug Patterns in the NCI-60 Cell Line Set. Cancer Res..

[16] Chakraborty A.R., Robey R.W., Luchenko V.L., Zhan Z., Piekarz R.L., Gillet J.-P., Kossenkov A.V., Wilkerson J., Showe L.C., Gottesman M.M. (2013). MAPK Pathway Activation Leads to Bim Loss and Histone Deacetylase Inhibitor Resistance: Rationale to Combine Romidepsin with an MEK Inhibitor. Blood.

[17] Sivanand S., Viney I., Wellen K.E. (2018). Spatiotemporal Control of Acetyl-CoA Metabolism in Chromatin Regulation. Trends Biochem. Sci..

[18] Pietrocola F., Galluzzi L., Bravo-San Pedro J.M., Madeo F., Kroemer G. (2015). Acetyl Coenzyme A: A Central Metabolite and Second Messenger. Cell Metab..

[19] Kurdistani S.K. (2014). Chromatin: A Capacitor of Acetate for Integrated Regulation of Gene Expression and Cell Physiology. Curr. Opin. Genet. Dev..

[20] Drazic A., Myklebust L.M., Ree R., Arnesen T. (2016). The World of Protein Acetylation. Biochim. Biophys. Acta.

[21] Lin R., Tao R., Gao X., Li T., Zhou X., Guan K.-L., Xiong Y., Lei Q.-Y. (2013). Acetylation Stabilizes ATP-Citrate Lyase to Promote Lipid Biosynthesis and Tumor Growth. Mol. Cell.

[22] Gansemer E.R., McCommis K.S., Martino M., King-McAlpin A.Q., Potthoff M.J., Finck B.N., Taylor E.B., Rutkowski D.T. (2020). NADPH and Glutathione Redox Link TCA Cycle Activity to Endoplasmic Reticulum Homeostasis. iScience.

[23] Lewis C.A., Parker S.J., Fiske B.P., McCloskey D., Gui D.Y., Green C.R., Vokes N.I., Feist A.M., Vander Heiden M.G., Metallo C.M. (2014). Tracing Compartmentalized NADPH Metabolism in the Cytosol and Mitochondria of Mammalian Cells. Mol. Cell.

[24] Sun S., Han Y., Liu J., Fang Y., Tian Y., Zhou J., Ma D., Wu P. (2014). Trichostatin A Targets the Mitochondrial Respiratory Chain, Increasing Mitochondrial Reactive Oxygen Species Production to Trigger Apoptosis in Human Breast Cancer Cells. PLoS ONE.

[25] Zhang Y., Ishida C.T., Ishida W., Lo S.-F.L., Zhao J., Shu C., Bianchetti E., Kleiner G., Sanchez-Quintero M.J., Quinzii C.M. (2018). Combined HDAC and Bromodomain Protein Inhibition Reprograms Tumor Cell Metabolism and Elicits Synthetic Lethality in Glioblastoma. Clin. Cancer Res..

[26] Kaisar M.M.M., Pelgrom L.R., van der Ham A.J., Yazdanbakhsh M., Everts B. (2017). Butyrate Conditions Human Dendritic Cells to Prime Type 1 Regulatory T Cells via Both Histone Deacetylase Inhibition and G Protein-Coupled Receptor 109A Signaling. Front. Immunol..

[27] Silva M.F.B., Jakobs C., Duran M., de Almeida I.T., Wanders R.J.A. (2001). Valproate Induces in Vitro Accumulation of Long-Chain Fatty Acylcarnitines. Mol. Genet. Metab..

[28] Coudé F.X., Grimber G., Pelet A., Benoit Y. (1983). Action of the Antiepileptic Drug, Valproic Acid, on Fatty Acid Oxidation in Isolated Rat Hepatocytes. Biochem. Biophys. Res. Commun..

[29] Wardell S.E., Ilkayeva O.R., Wieman H.L., Frigo D.E., Rathmell J.C., Newgard C.B., McDonnell D.P. (2009). Glucose Metabolism as a Target of Histone Deacetylase Inhibitors. Mol. Endocrinol..

[30] Nguyen T.T.T., Zhang Y., Shang E., Shu C., Torrini C., Zhao J., Bianchetti E., Mela A., Humala N., Mahajan A. (2020). HDAC Inhibitors Elicit Metabolic Reprogramming by Targeting Super-Enhancers in Glioblastoma Models. J. Clin. Investig..

[31] Lee T.-I., Kao Y.-H., Tsai W.-C., Chung C.-C., Chen Y.-C., Chen Y.-J. (2016). HDAC Inhibition Modulates Cardiac PPARs and Fatty Acid Metabolism in Diabetic Cardiomyopathy. PPAR Res..

[32] Yang J., Jin X., Yan Y., Shao Y., Pan Y., Roberts L.R., Zhang J., Huang H., Jiang J. (2017). Inhibiting Histone Deacetylases Suppresses Glucose Metabolism and Hepatocellular Carcinoma Growth by Restoring FBP1 Expression. Sci. Rep..

[33] Davidson S.M. (2016). Environment Impacts the Metabolic Dependencies of Ras-Driven Non-Small Cell Lung Cancer. Cell Metab..

[34] Guillaumond F., Bidaut G., Ouaissi M., Servais S., Gouirand V., Olivares O., Lac S., Borge L., Roques J., Gayet O. (2015). Cholesterol Uptake Disruption, in Association with Chemotherapy, Is a Promising Combined Metabolic Therapy for Pancreatic Adenocarcinoma. Proc. Natl. Acad. Sci. USA.

[35] Carrer A., Trefely S., Zhao S., Campbell S.L., Norgard R.J., Schultz K.C., Sidoli S., Parris J.L.D., Affronti H.C., Sivanand S. (2019). Acetyl-CoA Metabolism Supports Multistep Pancreatic Tumorigenesis. Cancer Discov..

[36] Padanad M.S., Konstantinidou G., Venkateswaran N., Melegari M., Rindhe S., Mitsche M., Yang C., Batten K., Huffman K.E., Liu J. (2016). Fatty Acid Oxidation Mediated by Acyl-CoA Synthetase Long Chain 3 Is Required for Mutant KRAS Lung Tumorigenesis. Cell Rep..

[37] O’Farrell M., Heuer T., Grimmer K., Crowley R., Waszczuk J., Fridlib M., Ventura R., Rubio C., Lai J., Buckley D. (2016). Abstract LB-214: FASN Inhibitor TVB-2640 Shows Pharmacodynamic Effect and Evidence of Clinical Activity in KRAS-Mutant NSCLC Patients in a Phase I Study. Cancer Res..

