# Supplementary material for: A Histone Deacetylase Inhibitor Induces Acetyl-CoA Depletion Leading to Lethal Metabolic Stress in RAS-Pathway Activated Cells
Source: Cancers (Basel). 2022 May 26;14(11):2643. doi: 10.3390/cancers14112643 (PMC9179484; doi:10.3390/cancers14112643)

Original images for Figure S2a

WB ac-hist H3

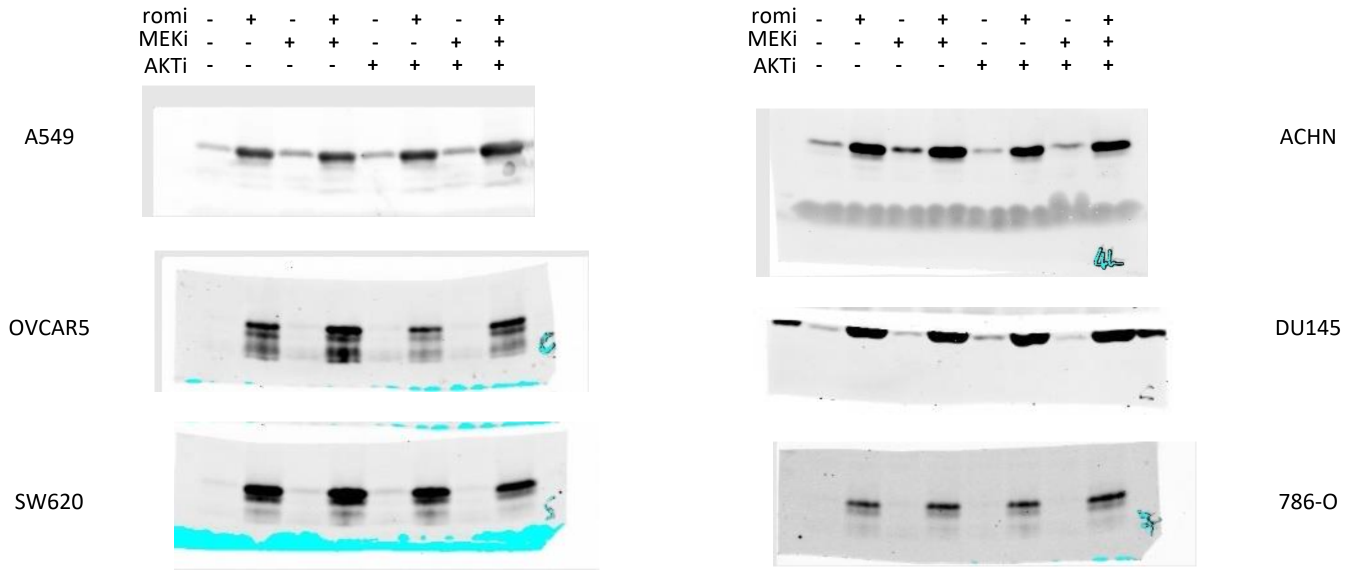

WB y-H2AX

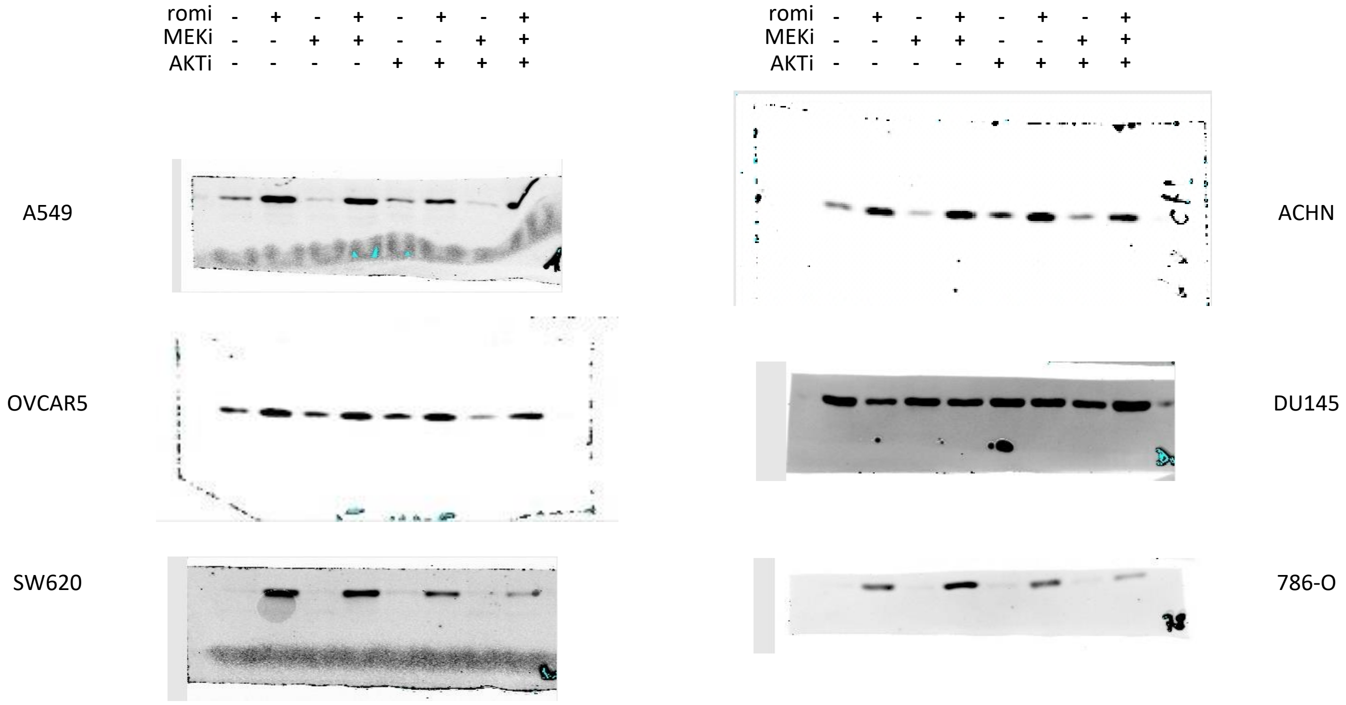

Original images for Supplementary Figure S8

WB ac-hist H3

+ romi

hour after treat. start 0 4 6 8 10 12 14 16 18

A549

ACHN

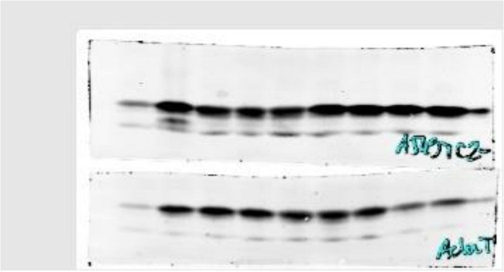

WB hist H3

+ romi

0 4 6 8 10 12 14 16 18

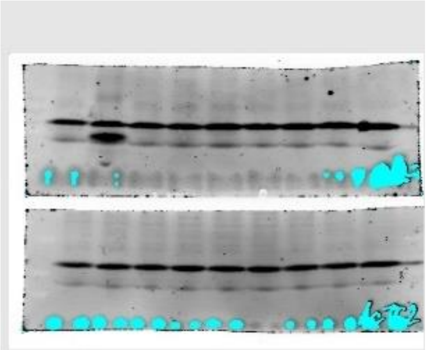

Supplement: Supplementary file 1 [file cancers-14-02643-s001.zip › Original_Images_for_Blots.pdf]
